# Incorporation of Mycobacteriophage Fulbright into Polycaprolactone Electrospun Nanofiber Wound Dressing

**DOI:** 10.3390/polym14101948

**Published:** 2022-05-11

**Authors:** Hari Kotturi, Charmaine Lopez-Davis, Sadegh Nikfarjam, Cameron Kedy, Micah Byrne, Vishal Barot, Morshed Khandaker

**Affiliations:** 1 Department of Biology, University of Central Oklahoma, Edmond, OK 73034, USA; snikfarjam@uco.edu (S.N.); ckedy@uco.edu (C.K.); mbyrne3@uco.edu (M.B.); 2 Department of Engineering and Physics, University of Central Oklahoma, Edmond, OK 73034, USA; vbarot@uco.edu

**Keywords:** antimicrobial agents, bacteriophages, electrospinning: mycobacterium smegmatis, polycaprolactone, mycobacteriophages

## Abstract

The Genus Mycobacterium includes pathogens known to cause disease in mammals such as tuberculosis (*Mycobacterium tuberculosis*) and skin infections (*M. abscessus*). *M. smegmatis* is a model bacterium that can cause opportunistic infections in human tissues and, rarely, a respiratory disease. Due to the emergence of multidrug-resistant bacteria, phage therapy is potentially an alternative way of treating these bacterial infections. As bacteriophages are specific to their bacterial host, it ensures that the normal flora is unharmed. Fulbright is a mycobacteriophage that infects the host bacteria *M. smegmatis*. The main goal of this study is to incorporate Mycobacteriophage Fulbright into a polycaprolactone (PCL) nanofiber and test its antimicrobial effect against the host bacteria, *M. smegmatis*. Stability tests conducted over 7 days showed that the phage titer does not decrease when in contact with PCL, making it a promising vehicle for phage delivery. Antimicrobial assays showed that PCL_Fulbright effectively reduces bacterial concentration after 24 h of contact. In addition, when stored at −20 °C, the phage remains viable for up to eleven months in the fiber. Fulbright addition on the nanofibrous mats resulted in an increase in water uptake and decrease in the mechanical properties (strength and Young’s modulus) of the membranes, indicating that the presence of phage Fulbright can greatly enhance the physical and mechanical properties of the PCL. Cytotoxicity assays showed that PCL_Fulbright is not cytotoxic to Balbc/3T3 mouse embryo fibroblast cell lines; thus, phage-incorporated PCL is a promising alternative to antibiotics in treating skin infections.

## 1. Introduction

Bacteriophage therapy, which uses bacterial viruses (phages) to treat bacterial infections, has been around for almost a century. During 1915, an outbreak of severe hemorrhagic dysentery among French troops in Paris occurred, and Felix d’Herelle was assigned to investigate the outbreak. He made bacterium-free filtrates of the soldiers’ fecal samples and incubated them with *Shigella* strains isolated from patients. A portion of the mixture was spread on an agar medium to observe bacterial growth, and he observed the appearance of clear plaques. He presented his findings during the Academy of Sciences meeting in 1917. Soon after discovering bacteriophages, d’Herelle used phages to treat *Shigella dysenteriae* in 1919, which was most likely the first attempt to use bacteriophages therapeutically [1]. However, the very existence of phages was not truly confirmed until the invention of electron microscopy in the 1940s [2,3].

Unreliable experimental success and poor documentation of studies led to reduced efforts for further phage research; moreover, the biological nature of phages was poorly understood. Rudimentary technical practices and improper storage resulted in low phage titers and contamination. In addition, medical limitations during that time made it unfeasible to find methods of phage delivery to infection sites [2]. While Western medicine dismissed phage therapy, the former Soviet Union and Eastern Europe continued to study phage therapy and conducted trials to treat a range of infectious agents such as Staphylococcus, Pseudomonas, and *E. coli* [4,5].

During the 1900s, major causes of death were infectious diseases such as cholera, diphtheria, syphilis, and many others. By the mid-20th century, the use of antibiotics and better sanitation practices prolonged life expectancy and improved the quality of life. However, it did not take long for bacteria to adapt and evolve, and they became equipped with antibiotic resistance genes such as ß-lactams, aminoglycosides, chloramphenicols, and tetracycline [2]. Antibiotic resistance genes are dangerous especially for difficult-to-treat infections such as *Mycobacterium abscessus*, which is a nontuberculosis mycobacteria that can cause disease to the skin, soft tissue, and central nervous system [6,7]. In the United States alone, around 2.8 million multidrug-resistant infections occur annually resulting in over 35,000 deaths [8]. Skin and soft tissue infections (SSTIs) are common types of infections that affect approximately 14 million people annually in the United States [9,10,11]. It is imperative that alternative treatments to bacterial infections are studied to preserve the length and quality of human life.

Thus, recent studies have focused on three groups of antimicrobials as alternatives to antibiotics: bacterial cell wall hydrolase (BCWH), antimicrobial peptides (AMPs), and bacteriophage [12]. BCWH has some disadvantages because it cannot be used toward Gram-negative bacteria due to the outer membrane, and some Gram-positive pathogens are already resistant to lysozymes. AMPs are effective against a plethora of bacteria and fungi but are costly and require high, toxic doses. Bacteriophage are promising, since they are highly specific, can infect both Gram-positive and Gram-negative bacteria, and are low-cost [13]. The specificity of bacteriophage to their bacterial host also ensures that normal microorganisms that are already present on the skin and are beneficial to the body remain unharmed.

Bacteriophage therapy can be performed in a plethora of ways. Topical applications have shown success when a study conducted by the Wound Centers at St. Joseph’s Medical Center in Tacoma used *Staphylococcus aureus* phages to treat diabetic toe ulcers of nine patients. The phage solution was applied to the ulcers once a week, and all infections responded to the phage therapy, and the ulcers healed within approximately seven weeks [14]. However, liquid and semi-solid gel formulations may be expensive and inconvenient to transport and store. The United States Army investigated the use of bacteriophages for food and safety and determined that phages must be stored and shipped dry to reduce the size and weight. While drying methods such as lyophilization or freeze drying is feasible, this can be expensive and time consuming. Thus, a promising method of phage application on skin infections is through electrospinning [15].

Electrospinning is a common method used to produce nanofibers with a diameter in the range of 100 nm or less [16]. A polymer solution is supplied from a spinneret, and a droplet forms at the spinneret exit. An electrical field is applied to the solution by placing an electrode in it and a counter-electrode at a distance from the spinneret. The Maxwell electrical stress stretches the droplet, and a Taylor cone forms due to the repulsion of the molecular charges, resulting in the ejection of a charged jet [16,17]. The ejected jet extends in a straight line and undergoes vigorous whipping due to bending instabilities. The jet continues to stretch and dry as the solvent eventually evaporates, and the spun nanofibers are deposited on the counter electrode [16].

There are several studies that have shown success with materials incorporated in electrospun fiber. A study by Salam et al. (2021) designed an electrospun nanofiber-based Viroblock (VB)-loaded polyacrylonitrile (PAN)/zinc oxide (ZnO) hybrid nanocomposite for personal protective equipment (PPE) applications. Their results showed an antibacterial activity of 92.59% and 88.64% against *Staphylococcus aureus* and *Pseudomonas aeruginosa*, respectively [18]. Dowlath et al. (2021) electrospun rabbit hemorrhagic disease virus (RHDV) Virus Like Particles (VLPs) as an antigen carrier into a polyvinylpy-roolidone (PVP)-based nanofiber and successfully showed that all treatments that contained the VLP induced an antibody response [19]. A study conducted by Kazsoki et al. (2021) showed the potential of using formulated core–shell-type nanofibrous scaffolds loaded with dexpanthenol (shell) and acyclovir (core) as an alternative treatment of herpes labialis [20].

Polycaprolactone (PCL) has been widely used in a large range of biomedical applications such as wound healing, drug delivery, and bone regeneration [21,22,23,24,25]. PCL is a hydrophobic polyester that has high elasticity and slow biodegradability; moreover, PCL cannot be easily broken down by enzymes and microorganisms, making it a promising method of phage delivery [13]. PCL is an aliphatic polyester belonging to the poly-α-hydroxy group [26] and is mainly degraded through hydrolysis of ester bonds on its molecular chains, generating carboxylic acid and hydroxyl functional groups, which reduces the molecular weight and decreases the overall weight of the polymer. The biodegradation rate of PCL is affected by its molecular weight, molecular weight distribution, crystallinity, and hydrophobicity of PCL molecular chains [23,27]. Many studies have shown great success incorporating various materials with PCL and demonstrated antimicrobial properties. He et al. (2021) constructed a sandwich-like dressing approach, with the top layer consisting of hydrophobic PCL nanofibers, the middle layer containing AgNP-loaded PCL/Gel nanofibers, and the bottom layer composing of hydrophilic PCL/gel nanofiber. Compared with a commercial silver sulfadiazine dressing, the designed wound dressing showed competitive antimicrobial properties, lower cell toxicity, and accelerated wounds closure for mouse skin injury [28]. Hassan et al. (2021) incorporated hydroxyapatite (HAP) doped with different concentrations of silver ions into PCL, and their in vitro cell proliferation assays using human fibroblasts cell lone (HFB4) showed that cells not only grew while in contact with the fibers, but they were also spreading and adhering through the deep pores [29].

In this study, the bacteriophage Fulbright was incorporated into PCL electrospun nanofibers. We examined the stability, cytotoxicity, and antibacterial activity of Fulbright-incorporated nanofiber dressing. This study demonstrates the potential of incorporating Mycobacteriophages into wound dressings as a safe and effective alternative to antibiotics.

## 2. Materials and Methods

### 2.1. Media Preparation and Bacterial Strain

*Mycobacterium smegmatis* mc^2^155 that was provided by the Hatfull lab at the University of Pittsburgh (Pittsburgh, PA, USA) was grown in the standard 7H9 liquid medium complete (7H9 broth base, 0.2% glycerol, albumin dextrose catalase (ADC) (10%V/V), 1 mM calcium chloride (CaCl_2_)). The liquid cultures were incubated in a shaking incubator (Fisher Scientific # SHKE4450) at 37 °C. On solid media, the bacteria were grown on the standard 7H10 agar plates (0.5% glycerol, 0.2% dextrose, and 1 mM calcium chloride (CaCl_2_)). The bacteria grown on 7H10 agar plates were incubated at 37 °C. Then, 50 µg/mL of cycloheximide and 50 µg/mL of carbenicillin were added to the liquid culture medium and 7H10 agar plates to reduce contamination. Afterwards, 2X Middlebrook top agar (7H9 broth base, 0.8% agar) was diluted at a 1:1 ratio with 7H9 liquid medium neat (7H9 broth base, 0.2% glycerol, 1 mM calcium chloride (CaCl_2_) to make 1X Middlebrook top agar, which was used to plate the bacterial lawn. The phage lysate was diluted in phage buffer (pH 7.2, 10 mM Tris, 10 mM magnesium sulfate (MgSO_4_), 70 mM sodium chloride (NaCl), and 1 mM CaCl_2_ to quantify the phage. For the agar-overlay method, 10 µL of each dilution was added to 250 μL of *M. smegmatis* mc^2^155 bacteria and incubated for 10 min. After incubation, 4.5 mL of 1X Middlebrook top agar was added to the bacteria and phage mixture and transferred to a 7H10 agar plate. The plates were incubated at 37 °C and assessed for plaques.

### 2.2. Media Preparation and Cell Line

Balbc/3T3 mouse embryo fibroblast cell line (ATCC #CCL-163) was provided by the Farris lab of Oklahoma Medical Research Foundation (Oklahoma City, OK, USA) and were grown using Dulbecco’s Modified Eagle Medium (DMEM) (4500 mg/L glucose, L-glutamine, sodium bicarbonate) (D5796) supplemented with sodium pyruvate (25-000-Cl), and FBS (26140-079). A vial of Balbc/3T3 was thawed and quickly transferred to 10 mL of DMEM supplemented media and centrifuged to remove the dimethyl sulfoxide (DMSO). The supernatant was removed, and the cell pellet was re-suspended in fresh media. The cell solution was transferred to a T25 cell culture flask and incubated at 37 °C, 5%CO_2_. When 80% confluency was reached, the cells were trypsinized, centrifuged, and re-suspended in fresh media and were split ⅕ every 3–4 days.

### 2.3. Polycaprolactone Fiber Production

**Figure 1:**
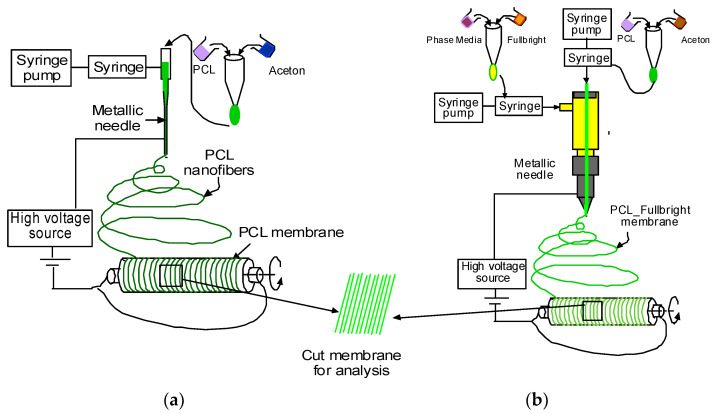
Schematic representation of the production of PCL and PCL_Fulbright nanofiber.

**Figure 2:**
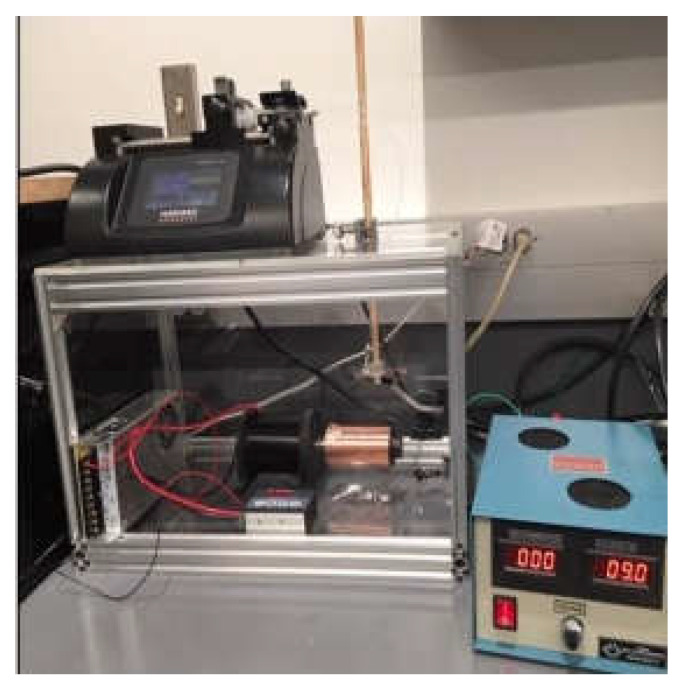
Electrospun fabrication unit used to produce the nanofiber dressing.

PCL and acetone solution mixture were prepared as described in our earlier research [30]. Figure 1a,b represents the schematic image of the PCL and PCL_Fulbright nanofiber membrane fabrication processes, respectively. PCL nanofiber membrane was produced by our custom-made electrospinning machine (Figure 2) using the PCL solution and a single axis one-inch discharge metallic needle (aluminum 23 G blunt needle, Model # BX 25). For the PCL_Fulbright membrane, a single-axis needle was replaced by a coaxial metallic needle (100-10-Coaxial-2822, Rame-hard Instrument Co., Succasunna, NJ, USA), which was attached with two syringe pumps to feed the PCL solution (core) and phage Fulbright suspended in phage buffer solution (shell) into a glass syringe and flowed through a tube to a metallic needle (Figure 2). The drum collector was spun using speed-controlled direct current (DC) motors. The syringe needle is electrically excited by applying a high voltage (9 kV) produced by a 0–30 kV high-voltage power supply (Model # ES 30, Gamma High Voltage Research Inc., Ormond Beach, FL, USA). This electrically charged syringe needle is positioned above a drum collector to capture the synthetic polymer fiber stream. We have optimized the speed of rotation, the distance between needle and drum, and the deposition rate of fiber on the drum to produce the fiber mat. The distance between the needle and drum collectors was approximately 5 cm. The feeding rate of the core and shell solutions will be adjusted to a rate of 0.025 mL/minute. The nanofiber was deposited on the drum with a 40 mm radius.

### 2.4. PEG Purification of Fulbright

For the preparation of our PCL_Fulbright nanofiber, polyethylene glycol (PEG 8000) purified phage lysate was used. The high titer phage lysate was centrifuged using a Thermo Scientific Sorvall Legend XTR centrifuge at 5500× *g* at 4 °C for 10 min. The supernatant was transferred to a new tube, and 1 M of NaCl and 10% of PEG were added. The solution was gently stirred at 15 rpm using a VWR Orbital Shaker at 4 °C overnight until the PEG and NaCl were in solution. The sample was then centrifuged at 5500× *g* at 4 °C for 10 min, and the supernatant was decanted, ensuring that most of the PEG has been removed. The pellet was gently re-suspended in fresh phage buffer and stirred overnight at 4 °C. Another centrifugation was performed, and the final cell pellet was re-suspended in phage buffer and stored at 4 °C until use. The phage titer in the PEG-purified lysate was 1 × 10^13^ PFU/mL. This lysate was used for all subsequent experiments unless otherwise noted.

### 2.5. Phage Stability and Storage Temperature

We examined the short-term and long-term effects of polycaprolactone on the stability of phage particles. For short-term effect, ≈2 cm^2^ of control PCL nanofiber was placed in a 5 mL phage lysate, and the tube was incubated at 37 °C. The control for this experiment was phage lysate without PCL fiber stored at 37 °C. The phage titer of the lysate soaked with control PCL was determined using standard agar-overlay methods each day for 7 days. For long-term effect, we tested the infectivity of the control PCL and PCL_Fulbright wound dressing stored at 4 °C and −20 °C at 7 days and 11 months post-production by testing the fiber for clear zones on a host bacterial lawn.

### 2.6. In Vitro Phage Release and Viability Assay

For the phage release assay, we followed the procedure described by Korehei and Kadla [31] with minor modifications. Briefly, ≈2 cm^2^ of PCL_Fulbright nanofiber was soaked in 3 mL phage lysate for 1 h with gentle shaking using a VWR Orbital Shaker. The soaked fiber was washed in 3 mL of phage buffer, which was shaken at 60 rpm at room temperature. The fibers were transferred into a fresh 3 mL phage buffer solution every 10 min for 10 washes. The PCL fiber was left to air dry for 2 h and stored at 4 °C overnight. The fibers were then plated on the host bacterial lawn and incubated at 37 °C for 48 h. The plates were assessed for clear borders surrounding the phage dressing.

A viability assay was performed to determine the number of phages released from the phage-incorporated fiber. The protocol outlined by Salalha et al. (2006) was followed with few modifications [16]. PCL_Fulbright nanofiber cut into ≈2 cm^2^ segments were placed in a microcentrifuge tube containing 1 mL of phage buffer. The tube was incubated for 60 min at room temperature to allow the phages to release into solution. After incubation, the tube was vortexed, and ten-fold dilutions of the buffer solution were plated using the agar-overlay method. The plates were incubated at 37 °C for 48 h, and PFU/mL was determined.

### 2.7. Antimicrobial Assay

To determine the antimicrobial activity of PCL_Fulbright, we followed the protocol outlined by Noguiera et al. (2017) [13]. The bacterial culture was adjusted to 1.5 × 10^8^ CFU/mL per McFarland standards using a Grant-bio Den-1 McFarland Densitometer. The bacteria were diluted to a final concentration of 1.5 × 10^6^ CFU/mL, and 1 mL of the bacteria was transferred into each culture tube. PCL and PCL_Fulbright cut into ≈2 cm^2^ were added to the culture tubes containing bacteria. Another tube containing bacteria alone served as a control. All the tubes were incubated at 37 °C for 24 h. After incubation, the bacterial solution in the culture tubes was diluted using ten-fold dilution and plated on 7H10 agar plates. The plates were incubated at 37 °C for 24 h. To determine the bacterial growth inhibition (% of Inhibition) at 24 h, the following calculation was used:

$$\%\;Inhibition=\frac{C-A}{C}\times 100$$

where *C* is the average value of Colony-Forming Units (CFU) of the controls, and *A* is the average value of CFU of PCL_Fulbright.

### 2.8. Direct Contact Cytotoxicity Assay

Cytotoxicity assays were performed using the protocol outlined by Nogueira (2017) [13] to determine the effect of PCL_Fulbright nanofiber on cell viability. Balbc/3T3 CCL-163 mouse embryo fibroblast cells were grown as recommended by the manufacturer. Cell viability was measured using AlamarBlue (ThermoFisher DAL 1100). Controls for this experiment were wells containing Balbc/3T3 cells alone and wells containing PCL plus cells. This experiment was conducted in quadruplets. For the direct contact assay, 500,000 Balbc/3T3 cells per well were seeded directly on ≈2 cm^2^ of PCL_Fulbright and PCL. and incubated at 37 °C, 5% CO_2_ for 24 h. After incubation, AlamarBlue was added as per the manufacturer’s recommendation. We transferred 100 µL from each well to a 96-well plate, and the absorbance was measured at 570 nm with a reference wavelength of 600 nm using a Biotek Synergy H1 Hybrid Reader. Random samples from each of the groups were randomly selected for DAPI staining.

### 2.9. DAPI Staining

Fibroblast cells attached to the wound dressing were visualized using fluorescent microscopy. 4′6-Diamidino-2-phenylindole (DAPI) is a commonly used fluorescent dye for staining DNA in cells. Random samples from the cytotoxicity assay were washed three times with phosphate buffer saline (PBS). After the final wash, 4% formalin was used to fix the cells. After fixation, the formalin was aspirated, and the fibers were washed with PBS three times. DAPI staining was performed in the dark at room temperature and was followed by five PBS washes prior to imaging. The morphology of the cell nuclei was observed using Zeiss Axiovert 200 m Inverted Fluorescent Microscope at 20x objective at an excitation wavelength of 350 nm.

### 2.10. Effect of Temperature on Phage Stability

The stability of phage Fulbright was evaluated at different temperatures (20 °C, 30 °C, 40 °C, 45 °C, 50 °C, 55 °C, 60 °C, and 65 °C), following the protocol described in our previous work [32]. A 1 mL volume of the phage lysate (1 × 10^12^ PFU/mL) was dispensed onto a 1.5 mL tube. The tubes were incubated at the above temperatures for 1 h. The temperature-subjected lysates were serially diluted in sterile phage buffer and then plated using the standard agar-overlay method. After 24 h of incubation, the number of PFU/mL was determined and plotted.

### 2.11. Effect of pH on Phage Stability

The stability of the virion particle was determined by incubating the phage in phage buffer adjusted to pH values (3, 4, 5, 6, 7, 8, and 9) for 1 h following the protocol described in our previous publication [32]. The pH of the phage buffer was adjusted using HCl or NaOH and was 0.22 µm filter-sterilized. The initial concentration of phage was 1 × 10^11^ PFU/mL, and it was incubated for 1 h at 37 °C. Following the incubation, phage was diluted, plated using the agar-overlay method, and incubated.

### 2.12. Scanning Electron Microscopy

Scanning electron micrographs of PCL and PCL_Fulbright were obtained using the Zeiss Neon 40 EsB. The samples were directly imaged at 50,000× with an accelerating voltage of 5 kV. The interaction between *M. smegmatis* and the PCL_Fulbright nanofiber was imaged using a Thermo Quattro S-Field Emission Environmental Scanning Electron Microscope at 5000× without any staining at an accelerating voltage of 20 kV.

### 2.13. Tensile Strength Assay

**Figure 3:**
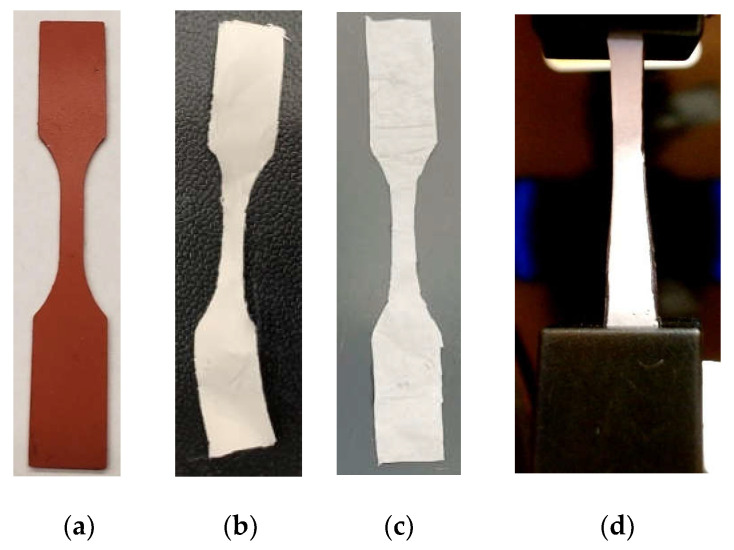
(**a**) ASTM D638 standard plastic specimen mold used to make tension test specimen. Prepared samples: (**b**) PCL and (**c**) PCL_Fulbright. (**d**) A sample in the gripper during the mechanical tests.

This study used a CellScale UniVert biomaterials testing machine (Waterloo, Ontario, Canada) to compare the tensile strength difference between PCL and PCL_Fulbright nanofibers. Three samples were prepared from a 15 cm × 20 cm piece of PCL and PCL_Fulbright membranes by placing an ASTM D638 standard plastic specimen mold (Figure 3a) and cutting along the edge of the mold using a razor blade. Figure 3b,c show the prepared PCL and PCL_Fulbright samples. The dimension of the gauge length section was 10 mm × 3 mm × 0.025 mm (length × width × thickness). Each sample was mounted on the grips (Figure 3d), and tension tests were conducted using a 100 N load cell with a 1 mm/min rate to each test sample. The load and displacement values were recorded from each experiment until the failure of the samples. The tension modulus and maximum tensile stress were calculated from the calculated stress and strain values from the recorded load and displacement values.

### 2.14. Water Absorption Test

Water absorption tests were conducted of according to a standard method described in detail in our earlier reference [33]. Three pieces of PCL and PCL_Fulbright membrane were cut from a fabricated mat, and we soaked each membrane in distilled water for 5 min. Each piece was air-dried for 30 min. Each sample weight before soaking (W_0_) and after air-dried (W_t_) was measured using a precision scale. The percentage of water absorption of each sample was measured using (W_t_ − W_0_) × 100%/W_0_ and compared.

### 2.15. Statistical Analysis

Graphpad prism was used to calculate one-way ANOVA and Tukey post hoc tests to establish multiple comparisons between samples. *p*-values below 0.05 were considered statistically significant. All experiments were conducted in triplicate and repeated 3 times unless stated otherwise.

## 3. Results

### 3.1. Phage Stability and Storage Temperature

**Figure 4:**
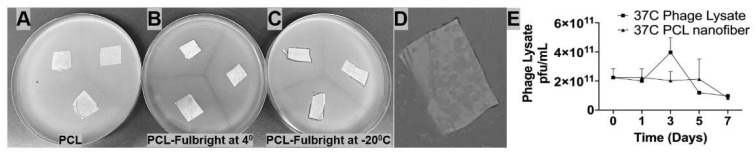
Panels A–D represent nanofibers stored at different temperatures and time periods. (**A**) PCL nanofiber at 4 °C stored for 7 days, (**B**) PCL_Fulbright at 4 °C for 7 days, (**C**) PCL_Fulbright at −20 °C stored for 7 days (**D**) PCL_Fulbright at −20 °C stored for 11 months. (**E**) Stability of phage particle with PCL nanofiber. All nanofibers were plated on *M. smegmatis* lawn. Arrows are pointing to a clear zone formed by phage lysis of host bacteria.

The short-term and long-term stability of the phage particle in the PCL_Fulbright nanofiber was examined in our experiments. The stability of Fulbright lysate in the presence of PCL was observed over the course of 7 days at 37 °C. We found no significant difference in phage titer between PCL fiber immersed in the phage lysate and the control phage lysate at 37 °C. *p* values were *p* = 0.9760 (37 °C vs. 37 °C with PCL) (Figure 4E). Our results (Figure 4A–D) indicate that the phage particle loses its infectivity when stored at 4 °C for 7 days, as indicated by the absence of a clear zone (Figure 4B). However, the wound dressings stored at −20 °C retain their antimicrobial activity at day 7 post-production (Figure 4C). There is still some antimicrobial activity against the host bacteria when stored at −20 °C, even after 11 months indicated by clearing (Figure 4D).

### 3.2. In Vitro Phage Release and Viability Assay

**Figure 5:**
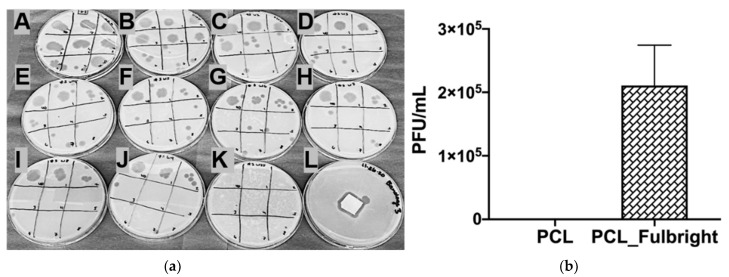
(**a**) In vitro phage release and (**b**) phage viability assay. (**a**) Each (**A**–**K**) represents the spot test of phage buffer after each wash. The spots on each plate represent the 10-fold dilutions of the phage buffer. (**A**–**J**) indicate the release of phage Fulbright from the nanofiber into the phage buffer. (**K**) indicates no residual phages in the phage buffer. (**L**) shows a clear zone around the washed nanofiber incubated on a lawn of host cells. (**b**) The graph panel represents the number of viable phage particles in the viability assay.

We examined the release of phage Fulbright from PCL_Fulbright electrospun fibers by submerging the PCL_Fulbright fiber in the phage buffer. We spot tested each of the 10-fold dilutions from each wash. Our results (Figure 5a) show that most of the phage is released in the first 2 h, as indicated by the presence of plaques on plates A through J and a lack of plaques on plate K. However, even with the overnight storage at 4 °C, the phage was still active and lysed the host bacteria. In the phage viability assay, the number of viable phages released per ≈2 cm^2^ of PCL_Fulbright fiber was quantified (Figure 5b). Our results indicate that soaking PCL_Fulbright nanofiber dressing in the phage buffer for 60 min resulted in the release of +/−2.1 × 10^5^ PFU/mL infectious particles from the fiber quantified using the standard agar-overlay method.

### 3.3. Antimicrobial Assay

**Figure 6:**
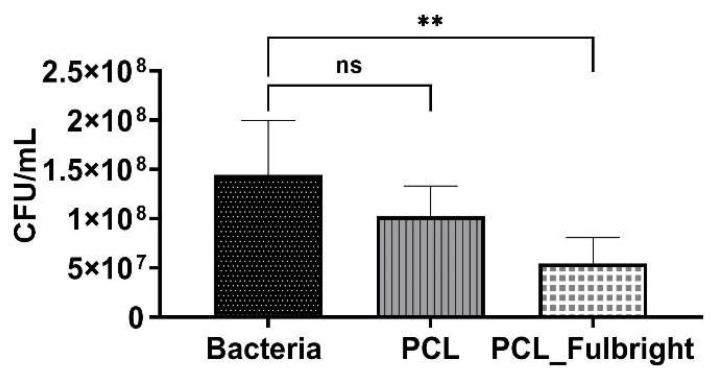
Antimicrobial activity of PCL_Fulbright against *M. smegmatis* at 24 h. ns = no significant difference and ** *p* value < 0.00.

The antimicrobial activity of PCL_Fulbright against *M. smegmatis* was measured at 4 h and 24 h of incubation with 1.5 × 10^6^ CFU/mL of the host bacteria. At 4 h of incubation, PCL_Fulbright was ineffective at decreasing *M. smegmatis* concentration (data not shown). However, after 24 h, there was a significant difference in bacterial concentration between bacterial control suspension without PCL fiber and PCL_Fulbright (Figure 6). The *p* values comparing the control and PCL_Fulbright were *p* = 0.0049. The percentage of inhibition was 62.93%.

### 3.4. Cytotoxicity Assays

**Figure 7:**
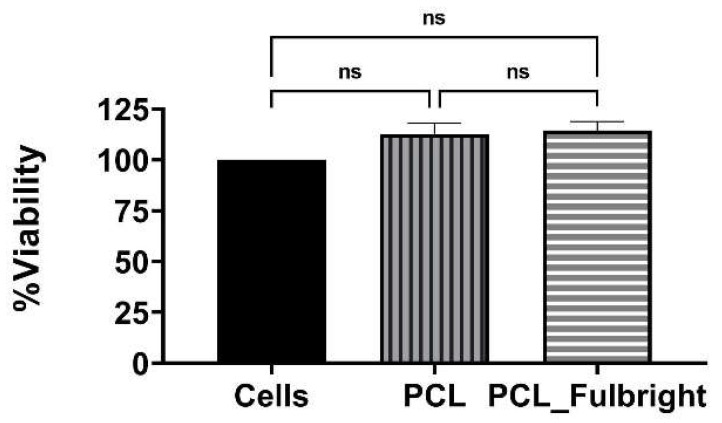
Cytotoxicity assay against Balbc/3T3 mouse embryo fibroblast cell line. ns = no significant difference.

**Figure 8:**
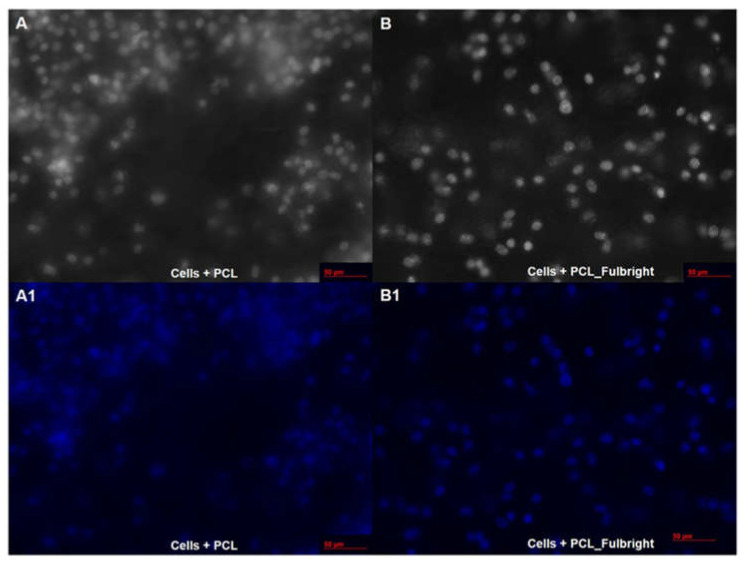
Fluorescent microscopy image of PCL dressing with mouse fibroblasts on top of the wound dressing in black and white (**A**) with DAPI filter (**A1**). (**B**,**B1**) represent cells on top of the phage incorporated PCL_Fulbright wound dressing.

Our cytotoxicity assay did not show any cytotoxic effect of PCL or PCL_Fulbright on the mouse 3T3 fibroblast cell line (Figure 7). The assay showed average values that did not range beyond 30% from controls. Per Noguiera (2017); only an alteration under or over 30% in comparison with controls would be considered cytotoxic or pro-tumorigenic, respectively [13]. The fluorescent microscopy image (Figure 8) of the cells attached to the PCL and PCL_Fulbright show nuclei with normal phenotype, further supporting that the phage particles in the PCL_Fulbright do not contribute to the cytotoxicity.

### 3.5. Thermal and pH Stability of Fulbright

**Figure 9:**
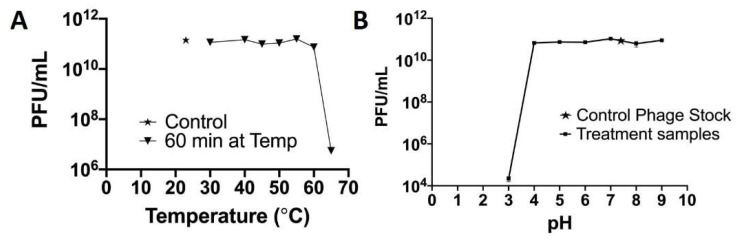
Temperature and pH stability of phage Fulbright.

There was no significant change in the infectivity of the phage between 20 and 55 °C. However, there was a 0.2 log reduction at 60 °C and a 4.3 log reduction in infectivity after 1 h at 65 °C (Figure 9A). At pH 3, there is a 5.69 log reduction in infectivity and with no significant change in the infectivity between 4 and 9 pH (Figure 9B).

### 3.6. SEM Imaging

**Figure 10:**
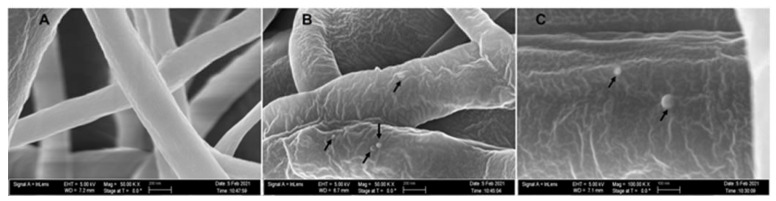
(**A**) represents PCL nanofiber. (**B**) is PCL_Fulbright with black arrows pointing to the phage particles. (**C**) is magnified at 100,000×.

**Figure 11:**
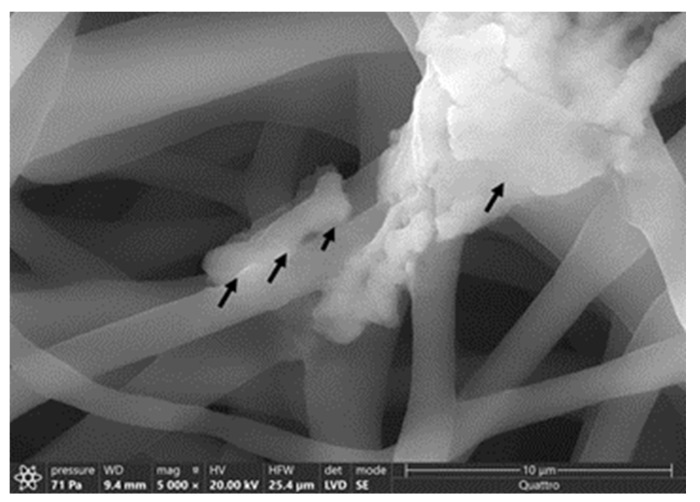
Interaction between *M. smegmatis* and PCL_Fulbright nanofiber. Black arrows are pointing to the bacterial cell attached to the nanofiber.

The average diameter of our nanofiber is ≈200 ± 96 nm. At 50,000× magnification, we can clearly see the incorporated phage particle in the PCL_Fulbright fiber represented by bright spherical spots (Figure 10B,C) as indicated by the arrows. We do not see those spots in the PCL fiber without phage (Figure 10A). We can see the random distribution of phage particles in the nanofiber core. The interaction between the PCL_Fulbright and the bacterial can be clearly seen in Figure 11. The phage particle was uniformly distributed along the length of the fiber. The distribution of phage particle on the fiber was measured from a SEM image. We estimated five phage particles per 500 nm along the fiber distance.

### 3.7. Mechanical Tests

**Figure 12:**
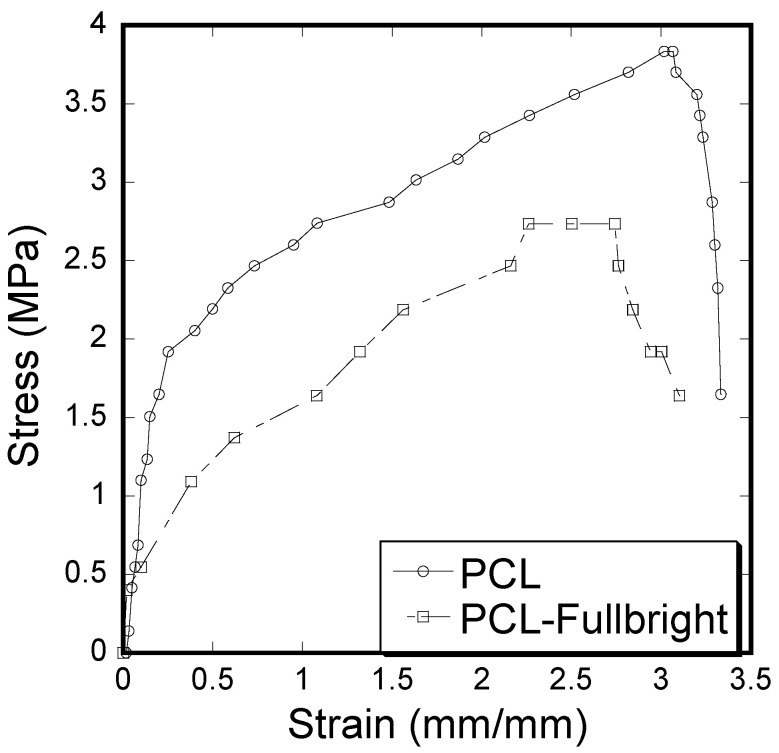
Stress–strain curve of pull-out tension tests showing the difference between the strain and stress for PCL and PCL_Fulbright cloths.

A clear difference of stress vs. strain curves (Figure 12) and mechanical properties (tensile modulus and strength) calculated from the stress vs. strain results (Table 1) between PCL and PCL_Fulbright samples was observed. Immobilization of Fulbright reduced the tensile strength and modulus significantly (*p* value < 0.05).

### 3.8. Water Absorption Tests

**Figure 13:**
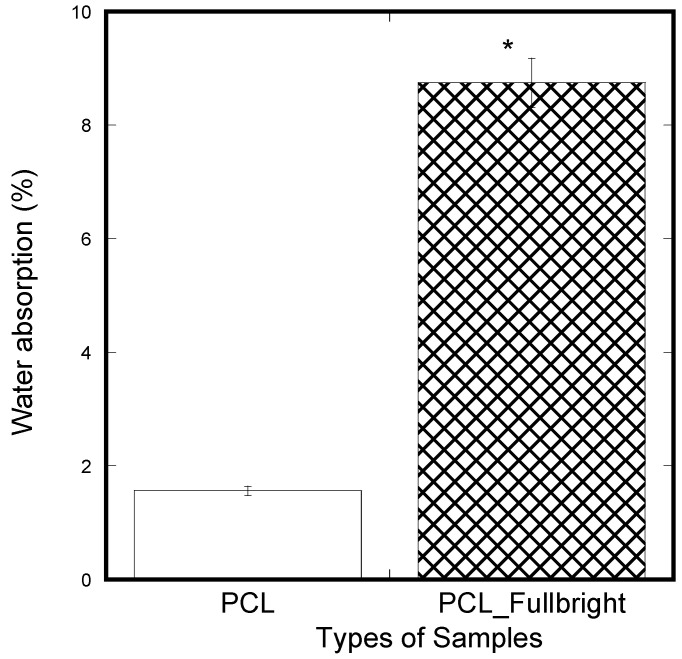
Weight percentage difference between the samples from the initial weight after applying soaking in water for 5 min and air-dried for 30 min. The values of all parameters were reported as mean ± SOE (n = 3). * refers *p* < 0.05 with respect to PCL.

A significantly lower amount of water absorption was observed in the PCL membranes compared to the PCL_Fulbright membranes (Figure 13). In our previous study using two different thickness of PCL cloths, we found a negligible water absorption on both PCL cloths. The results mean that phage Fulbright attachment with the PCL fiber makes PCL fiber more hydrophilic.

## 4. Discussion

Bacteriophages are highly specific and only infect and kill their host bacteria. This quality makes them ideal for treating antibiotic-resistant bacterial skin infections, as they can leave the normal microbiota and human tissues intact. We incorporated a Mycobacteriophage Fulbright into polycaprolactone (PCL) nanofiber dressing, evaluated its stability in the nanofiber matrix, phage release from the nanofiber, its antimicrobial effect on host bacteria, and its cytotoxicity against a mouse fibroblast cell line, Balbc/3T3.

We have evaluated the stability of phage Fulbright in two ways. First, we examined the stability of the particle in contact with PCL, and second, we also assessed its stability when incorporated into the PCL nanofiber. Our results show that there is no significant change in the phage titer when it is in contact with PCL fiber (Figure 4E) for up to 7 days. When the phage is incorporated into the PCL nanofiber, the ideal storage temperature to retain the phage viability is −20 °C, as we lost the activity after a week when stored at 4 °C. We were able to detect phage activity at −20 °C for up to 11 months. Our results agree with published studies by Nogueira (2017) in terms of PCL being an excellent medium for phage delivery [13]. Studies conducted by Koo (2016) further support the effect of storage temperatures on the infectivity of bacteriophages in electrospun nanofibers with higher phage activity at −20 °C [15]. These observations are also confirmed by Salhala (2016) and Korehei (2014) [16,31].

In 2014, Korehei and Kadla (2014) investigated the effects of electrospinning bacteriophage T4 in poly (ethylene oxide) and cellulose diacetate fibers [31]. A rapid release of T4 was observed once suspended in an aqueous buffer medium. A release of 100% T4 phage occurred within 30 min of immersion due to the high hydrophilicity of PEO. Our in vitro phage release assay demonstrates that when soaked in phage buffer for about 2 h, an average of 2.11 × 10^5^ PFU/mL of Fulbright was released per 2 cm^2^ of nanofiber. However, the wound dressing retained its antimicrobial activity indicated by a clear zone on a lawn of host bacteria (Figure 5a, Plate L). The ability of the wound dressing to maintain a slow and sustained release such as ours may be an ideal trait for treating infections caused by slow-growing bacteria.

The antimicrobial activity of PCL_Fulbright wound dressing in contact with *M. smegmatis* culture at 4 h of contact showed no reduction in bacterial concentration. This may be due to the hydrophobic characteristic of PCL fiber and mycolic acid in the *M. smegmatis* cell wall. Previous studies by Yang and Deng demonstrated a reduced adhesion capacity of PCL to bacteria [34]. However, at 24 h, there is a percentage inhibition of 62.93%, indicating that once the phages are released into the media, the phages lysed the host cells successfully. We tested the cytotoxic effect of PCL_Fulbright on a mouse fibroblast cell line. Our results indicate that PCL_Fulbright does not have any cytotoxic effect on cells. The direct contact assay showed an average growth enhancement of 14.39% in the presence of phage-incorporated wound dressing. Our previous studies using fibroblasts, osteoblasts, and mesenchymal stem cells with PCL nanofiber also support this observation [35,36,37].

As phage Fulbright is subjected to temperature fluctuations during the production of PCL_Fulbright nanofiber, we also examined the infectivity of the phage particles at various temperatures and pHs (Figure 8). The phage particle is infective up to 60 °C, and there is a 4.3-log reduction at 65 °C. For pH, it appears that highly acidic pH (≤3) disrupts the structure of phage particle. Previous studies have shown that the inability of the phage to form plaques at higher temperatures, highly acidic, and highly basic environments is due to the denaturation of phage and host proteins [38]. These data are consistent with our previous work with other Mycobacteriophages in terms of their stability [32,37,39,40].

The stress vs. strain behavior due to tensile force (Figure 12) of PCL_Fulbright fibrous membranes was compared with that of pure PCL membrane. As shown in Table 1, PCL fibrous membranes exhibit the higher tensile strength and modulus compared to the values of PCL_Fulbright. The tensile strength values of PCL (5.28 ± 0.11 MPa) were in agreement with the PCL membranes strength found by Cheng et al. The decrease in mechanical properties due to the incorporation of bacteriophages in PCL can be justified with the fact that the wt % of PCL in PCL_Fulbright is less compared to the PCL. The study by Cheng (2018) found that the increase in PCL concentration in PCL with bacteriophages increased the tensile strength [41].

Since water absorption ability is an important parameter for cell adhesion, water absorption tests were performed on the nanofibrous mats. The water absorption of PCL_Fulbright fibrous membranes was compared with that of pure PCL membrane. The result indicated that PCL_Fulbright nanofibers have higher water uptake than PCL nanofibrous mats. The water uptake of PCL_Fulbright compared to PCL nanofiber structures is due to the hydrophilicity of phage Fulbright and morphological differences between the two samples. The higher water absorption of the PCL_Fulbright membrane observed compared to PCL agrees with previous research [42,43] where it is reported that the presence of protein and porosity might be mainly responsible for the difference between total water uptake values of PCL fiber mats.

Presently, there are commercial antimicrobial applications that have limitations, such as Dermasilk^®^, which is a silk-based material with Silane quaternary ammonium compounds (Si-QAC). Padycare^®^ and TheraBond^®^ take advantage of Silver’s antimicrobial properties. Unfortunately, Si-QAC has been reported to cause bacterial resistance and skin sensitization [13]. Although silver has been heavily used as antimicrobial dressings in managing the wound infections of patients, there is a lack of knowledge regarding it effects on the normal healing process of the body [44]. Silver can be toxic to fibroblasts and keratinocytes at high concentrations [45]. Nesporova (2020) concluded that silver dressings should only be used in highly infected wounds, as sub-toxic levels of silver could induce intracellular ROS production and DNA breaks, affecting rapidly proliferating cells during the wound-healing process [12].

The limitation of this study is that we did not conduct contact an analysis to measure the sample groups’ hydrophobicity. Our previous study on contact angle measurement tests on the various thickness of PCL membranes found that PCL membranes are very hydrophobic (contact angle values varied between 120 and 150 degrees) [30]. The contact angle generally shows a linear relation to the absorbed water drop volume, independent of the substrate water absorption rate, drop volume, and contact time [46]. This means that a linear decrease in the measured contact angle can be observed with increasing water absorbed drop volume. We have observed a 5.6 times rise in water absorption value due to the incorporation of phage Fulbright with PCL; therefore, a decrease in the contact angle value of Fulbright_PCL is anticipated. Considering a linear relationship between water contact angle and absorbed drop volume, we expect that Fulbright_PCL is still regarded as hydrophobic, since the water contact angle above 90 degrees is considered hydrophobic. Our future study will confirm the hydrophobicity of the Fulbright_PCL sample.

## 5. Conclusions

This study aims to incorporate Mycobacteriophage Fulbright into a polycaprolactone (PCL) nanofiber and test its antimicrobial effect against the host bacteria. We successfully combined PCL’s biocompatibility and enhanced the nanofiber’s antimicrobial properties against mycobacterial infections by incorporating mycobacteriophages. Our produced phase_PCL nanofiber membranes have the potential for phage therapy trials to treat skin, bloodstream, urinary tract, and other soft tissues infections.

## Figures and Tables

**Table 1:** Mechanical test results of PCL and PCL_Fulbright samples. The data are presented as mean ± standard of error for sample size, n = 3.

Sample Number	Tensile Strength		Tensile Modulus	
	PCL	PCL_Fulbright	PCL	PCL_Fulbright
1	5.37	2.45	9.22	3.74
2	5.47	3.28	13.67	5.48
3	5.01	2.73	9.58	1.89
Average	5.28	2.82	10.82	3.70
St dev	0.11	0.20	1.17	0.85
*p* value		0.00		0.02

## Data Availability

Not applicable.
